# Prevalence of pain and its socio-demographic and clinical correlates among heroin-dependent patients receiving methadone maintenance treatment

**DOI:** 10.1038/s41598-017-09404-w

**Published:** 2017-08-18

**Authors:** Ying-Jia Yang, Yan-Min Xu, Wen-Cai Chen, Jun-Hong Zhu, Jin Lu, Bao-Liang Zhong

**Affiliations:** 1 Shenzhen Key Laboratory for Psychological Healthcare, Shenzhen Institute of Mental Health, Shenzhen Kangning Hospital, Shenzhen Mental Health Center, Shenzhen, Guangdong Province China; 2 Affiliated Wuhan Mental Health Center (The Ninth Clinical School), Tongji Medical College of Huazhong University of Science & Technology, Wuhan, Hubei Province China; 3 Department of Psychiatry, The First Affiliated Hospital of Kunming Medical University, Kunming, Yunnan Province China

## Abstract

To date there have been no studies investigating the characteristics of pain in Chinese heroin-dependent patients (HDPs) receiving methadone maintenance treatment (MMT). This study examined the frequency and socio-demographic and clinical correlates of pain in HDPs under MMT. A consecutive sample of 603 HDPs was recruited from three MMT clinics in Wuhan, China. These patients completed a standardized questionnaire concerning socio-demographic and clinical data. Pain intensity was assessed with the 5-point Verbal Rating Scale (“Overall, how intense is your pain now?”) with responses of: 1 = none, 2 = mild, 3 = moderate, 4 = severe, 5 = very severe. A pain score of three or higher was used to denote clinical significant pain (CSP). The prevalence of CSP in HDPs receiving MMT was 53.6%. Factors significantly associated CSP in multiple logistics regression analysis were old age, marital status of “non-married”, unemployment, having religious beliefs, a history of injecting heroin, a high dose of methadone, and more depressive symptoms. Over a half of Chinese HDPs receiving MMT have CSP. Services for HDPs in MMT settings should include periodic screening for pain, psychosocial supports, and professional treatment for pain.

## Introduction

The relationship between pain and substance dependence is a complex and important clinical issue^1^. Studies have demonstrated that pain is very common among patients with various types of drug dependence disorders^2–4^, and chronic pain is associated with the development and relapse of opioid addiction, and craving for opioids^5, 6^. Further, studies also found that chemically dependent patients with clinically significant pain (CSP) are more likely to self-medicate with other illicit drugs, alcohol, and non-prescribed drugs^2, 7–10^. Importantly, drug-dependent patients are commonly under-treated for pain because their requests for analgesia are often misunderstood as craving for drugs, and physicians who treat patients with chronic pain are grappling with how to deal with drug dependence^11, 12^. These potential negative consequences highlight the need for studies that evaluate the prevalence and characteristics of pain among drug-dependent patients.

In China, methadone maintenance treatment (MMT) is an effective national strategy to reduce the harms of opioid (mainly heroin) use such as drug-related criminal activities, and address its related public health issues such as HIV and HCV epidemic^13–16^. The MMT programs are now rapidly expanding in China. According to official statistics, by 2013, a total of 412,686 heroin-dependent patients (HDPs) had enrolled in 763 MMT clinics distributed in nearly all provinces of China^17^. Although there is evidence that MMT significantly improves the social well-being and quality of life (QOL) of Chinese HDPs^18, 19^, studies still reported, compared to healthy controls, a significantly poorer QOL in Chinese HDPs receiving MMT^20^. A further analysis on related factors of QOL revealed that pain significantly contributed to the poor physical and mental QOL of Chinese HDPs under MMT^21^.

Data on the epidemiology of pain in Chinese HDPs are very limited. Only two recent studies reported that the prevalence rates of chronic severe pain in HDPs of one compulsory drug rehabilitation center and CSP in HDPs of MMT clinics were 39.7% and 14.1%^21, 22^, respectively. Of the two studies, one had analyzed the characteristics of HDPs with chronic severe pain^22^, however, because this study recruited a small sample of HDPs from one compulsory drug rehabilitation center only, the generality of its findings is very limited. Understanding the prevalence and correlates of pain in Chinese MMT settings would lend additional insights into the complex pain-addiction relationship, as well as the management of pain in HDPs. This study was carried out to determine the prevalence and characteristics of pain in Chinese HDPs treated in MMT clinics. The hypothesis of this study was that pain would be prevalent in Chinese HDPs, and it would be associated with a number of socio-demographic and clinical characteristics.

## Results

A total of 603 of 652 (92.5%) eligible patients completed the survey. The average age of the 603 subjects was 38.1 years (standard deviation [SD] = 7.0, range = 21–59), and 68.3% were men. Most patients (84.1%) injected heroin before being admitted to MMT, and the mean dose and duration of methadone were 69.5 mg/d (SD = 29.6) and 24.6 months (SD = 11.0), respectively.

In total, 323 HDPs endorsed CSP. The prevalence of CSP was 53.6% in the whole sample, with 54.1% in men and 52.4% in women. Results of the comparison between no pain (NP) and CSP groups (Table 1) showed that, compared to NP group, HDPs of CSP group were significantly more likely to be older, have an educational attainment of primary school and below, be non-married, be unemployed, have religious beliefs, currently smoke, have a history of intravenous heroin use, take a high dose of methadone, and have more depressive symptoms.

**Table 1:** Comparison of socio-demographic and clinical characteristics between methadone patients with and without pain.

Characteristics	No pain group (N = 280)	Clinically significant pain group (N = 323)	Statistics	P
Gender: male^#^	189(67.5)	223(69.0)	χ2 = 0.205	0.651
Age (years)^&^	36.9(6.9)	39.2(7.0)	t = 3.942	<0.001
Education: primary school and below^#^	20(7.1)	44(13.6)	χ2 = 5.670	0.017
Marital status: non-married^*#^	124(44.3)	184(57.0)	χ2 = 8.974	0.003
Unemployment^#^	134(41.5)	198(70.7)	χ2 = 15.458	<0.001
Reporting religious beliefs^#^	14(5.0)	72(22.3)	χ2 = 33.601	<0.001
Currently smoking^#^	240(85.7)	312(96.6)	χ2 = 25.503	<0.001
A history of injecting heroin^#^	223(79.6)	284(87.9)	χ2 = 6.805	0.009
Duration of heroin use (years)^&^	9.7(4.1)	10.2(4.3)	t = 1.644	0.101
Methadone dosage (mg/day)^&^	65.5(29.1)	72.8(29.7)	t = 2.918	0.004
Duration of methadone maintenance treatment^&^	24.7(11.6)	24.6(10.4)	t = 0.071	0.943
Self-rating Depression Scale^&^	36.5(6.3)	40.2(9.5)	t = 5.487	<0.001

^*^“Non-married” includes never-married, remarried, cohabitating, separated/divorced, and widowed.

^#^Categorical variables are expressed as number of cases (%).

^&^Continuous variables are expressed as mean (standard deviation).

Multiple logistic regression (Table 2) revealed that old age, marital status of “non-married”, unemployment, having religious beliefs, a history of injecting heroin, a high dose of methadone, and more depressive symptoms were significantly associated with CSP.

**Table 2:** Multivariate logistic regression results of factors significantly associated with clinically significant pain.

Factor	Risk level	Reference level	Coefficient	Standard error	Wald χ2	P	OR(95%CI)
Age (years)^&^	—	—	0.099	0.016	37.51	<0.001	1.10(1.07,1.14)
Marital status	Non-married*	Married	1.120	0.223	25.165	<0.001	3.07(1.98,4.75)
Unemployment	Yes	No	0.646	0.206	9.807	0.002	1.91(1.27,2.86)
Presence of religious beliefs	Yes	No	1.558	0.382	16.675	<0.001	4.75(2.25,10.03)
Usual route of heroin administration before MMT	Injecting	Smoking	0.915	0.282	10.515	0.001	2.50(1.44,4.35)
Methadone dosage (mg/day)^&^	—	—	0.007	0.004	4.182	0.041	1.02(1.01,1.03)
Self-rating Depression Scale^&^	—	—	0.043	0.013	11.708	0.001	1.04(1.02,1.07)

^&^Continuous variables, the ORs for 1 unit increase in age (a year), methadone dose (1 mg/day), and Self-rating Depression Scale score (1 point) are 1.10, 1.02, and 1.04, respectively.

*“Non-married” includes never-married, remarried, cohabitating, separated/divorced, and widowed.

## Discussion

To the best of our knowledge, this is the first detailed report on the epidemiology of pain in Chinese HDPs in MMT settings. In the present study, the estimated prevalence of CSP was 53.6% among HDPs under MMT. Indeed, it is difficult to directly compare our pain prevalence with that of previous studies due to various measures and definitions of pain and samples with various substance use disorders. Nevertheless, the high prevalence of pain in HDPs we found is concordant with findings reported in other studies, including the 37% prevalence of chronic severe pain in patients from MMT programs^2^, the 24% prevalence of chronic severe pain in inpatients from short-term residential substance abuse treatment programs^2^, the 87% prevalence of chronic pain in primary care patients who were screened positive for any illicit drug use or prescriptions drug misuse^7^, the 29.1% prevalence of chronic severe pain in patients attending an outpatient drug and alcohol treatment program^23^, and the 55.3% prevalence of chronic pain in patients from a MMT clinic^24^.

The high prevalence of pain could be primarily due to the long-term heroin use of HDPs, because experimental studies have found that long-term use of opioids can lead to a phenomenon known as “opioid induced hyperalgesia” and an increase in the sensitivity to pain^25, 26^. In addition, the endogenous opioid system plays an important role in relieving pain of humans, for example, the human body manufactures opiate-like substances to provide pain relief at specific receptor sites in the central nervous system^27^. The long-term intake of exogenous opioids may have impaired body’s innate pain-relieving system of HDPs^28^.

Although several possible biological mechanisms may explain the observed high prevalence of pain in HDPs, our multiple regression analysis showed that pain was related to certain socio-demographic variables, characteristics of past opioid use and current MMT, and depressive emotion, indicating the complexity of pain in HDPs. Consistent with findings from one general population-based survey^29^ and two studies with patients from substance use disorders treatment modalities^2, 9^, we found a significant association between old age and CSP in HDPs. Previous research seldom studied the relationship between pain and marital status in drug-dependent patients^2, 9^, the significant contribution of non-married status on CSP of HDPs replicated findings from a survey on neuropathic pain of patients with traumatic brachial plexus injury^30^, which found marital status was an independent predictor of pain.

Pain is defined as “an unpleasant sensory and emotional experience associated with actual or potential tissue damage, or described in terms of such damage”^31^. Because pain is a subjective phenomenon, psychosocial factors may affect one’s experience of pain. In this case, it is reasonable to find non-married status, religious beliefs, and depression as significant risk factors of CSP in HDPs. For example, social support, including support from spouses of married individuals may buffer against the emotional distress induced by pain. Similarly, depression could exacerbate pain, as the depressive cognition of depressed patients may influence how HDPs perceive their pain^32^. Holding a religious belief is generally viewed as a protective factor against negative emotion in western countries^33, 34^. But in our study HDPs with religious beliefs had more intense pain than those without religious beliefs. It seems that holding religious beliefs is a risk factor for pain; this phenomenon is similar to some other studies in China that reported a significant association between the presence of religious beliefs and depression among internal medicine outpatients and individuals with visual disabilities^35, 36^. One possible explanation is that the majority of Chinese are atheists; individuals who subsequently become religious may do so because they are emotionally troubled by pain or other painful conditions. For example, over two-thirds Chinese female Buddhists have experienced at least one negative life event (i.e., serious physical illnesses, losing spouses, divorcement, and financial difficulties) before becoming Buddhists^37^.

Pain, in particular chronic pain, can interfere with daily activities and occupation, which in turn further results in permanent disability^2^. The significant link of unemployment with CSP in this study might be explained by the negative outcomes of CSP such as unemployment. Similarly, the significant association between a high dose of methadone and CSP in HDPs might be due to the analgesic effect of methadone: CSP HDPs need a high dose of methadone to relieve pain^24^. There is evidence that injection drug users are at very high risk for acquiring hepatitis C (HCV) and HIV/AIDS infection^38^, therefore, painful conditions such as Hepatitis, are common among HDPs with a history of injecting heroin^39, 40^, resulting in the significant link between injecting heroin and CSP.

The main limitation of this study is that this is a cross-sectional study so the correlates we found to be associated with pain are not, strictly speaking, risk factors. Whether or not these identified factors lead to pain need to be examined by prospective studies. A second limitation is that some other contributing factors of pain such as medical conditions, duration of treatment at the current methadone dose, and pain prior to heroin use, were not assessed in the study. Third, data on the type of pain, acute or chronic, are also clinically relevant, however, we did not assess the duration of patients’ pain. Fourth, although the high prevalence of pain in HDPs can be ascribed to the long-term use of heroin, our analysis did not ascertain the significant association between duration of past heroin use and CSP. In fact, pain should be related to the total amount of past exposure to heroin, which means that both the daily heroin dose and duration of heroin use should be taken into consideration when assessing pain’s association with past heroin exposure. Because duration of past heroin use is not a good proxy measure of past heroin exposure, we did not find a significant association between pain and duration of heroin use. Finally, pain was subjectively assessed in this self-report survey, so it is possible that some HDPs may deliberately exaggerate their pain intensity for the purpose of obtaining a higher dose of methadone.

In summary, the present study has demonstrated a high prevalence of CSP in HDPs receiving MMT in Wuhan, China. There is an urgent need for health workers of Chinese MMT clinics to address the epidemic of pain in HDPs. Efforts to relieve the pain of HDPs may be useful to target on those who are old, unmarried, unemployed, receiving a high dose of methadone, and depressed, as well as those who hold religious beliefs and have a history of injecting heroin. Psychosocial and medical services for HDPs in MMT clinics should include periodical assessment of pain, expanded social supports, and appropriate pain management.

## Methods

### Subjects

This was a secondary analysis of data from a cross-sectional survey, which investigated a range of health outcomes among HDPs of MMT clinics in Wuhan, Hubei Province, China^41^. We consecutively recruited HDPs from three city-owned MMT clinics between June 2009 and July 2010. Eligible subjects were patients aged 20 years or older and met DSM-IV diagnostic criteria for lifetime heroin dependence. Patients were excluded if they had severe physical illnesses, alcohol dependence, brain organic mental disorders, and psychotic symptoms.

The Institutional Review Board of Wuhan Mental Health Center approved the research protocol. The protocol including the methods was performed in accordance with the Declaration of Helsinki and the relevant ethical guidelines and regulations in China. Written informed consent was obtained from all participants.

### Survey instruments and procedures

This research was a self-administered questionnaire survey. Trained psychiatrists working in MMT clinics were arranged to read out questions for subjects who were illiterate or had difficulties in reading.

A questionnaire, specifically developed for this study, was used to collect socio-demographic and clinical data, including gender, age, education years, marital status, cigarette smoking, self-reported presence of religious beliefs, usual route of prior heroin administration (injecting or smoking), duration of heroin use, duration of MMT, daily methadone dosage at the time of the survey, and Zung’s Self-rating Depression Scale (SDS)^42, 43^. Current smokers were defined as those who currently smoked at least one cigarette per day on at least five days per week^44, 45^. The Chinese SDS is a 20-item self-report scale to assess the severity of depressive symptoms using a 4-point rating scale (1 = a little of the time to 4 = most of the time). Total SDS score varies between 20 and 80, with higher scores denoting more depressive symptoms.

Pain intensity was assessed with the 5-point Verbal Rating Scale (VRS), asking “Overall, how intense is your pain now?”; it has 5-category responses: 1 = None, 2 = Mild, 3 = Moderate, 4 = Severe, 5 = Very severe. The 5-point VRS measure of pain has been widely used in prior studies and has been shown to be as valid as other common measures of pain intensity^46, 47^. In accordance with previous studies^48, 49^, patients were classified as having CSP if they indicated their pain was “moderate”, “severe”, or “very severe”, while patients reported “none” or “mild pain” were classified as NP.

### Statistical analysis

Prevalence of CSP was calculated. Socio-demographic and clinical characteristics of CSP and NP groups were described and compared by t-test or Chi-square test, as appropriate. Multivariable logistic regression model with a backward stepwise entry of significant variables in the above univariate analysis was used to identify factors significantly associated with CSP. Odds ratios (ORs) and 95% confidence intervals (CIs) were used to quantify the associations between factors and CSP. The statistical significance level was set at p < 0.05 (two-sided). SPSS software version 16.0 package was used for analyses.
